# Chromic and dynamic: soft crystals of platinum(II) complexes pave the way for multi-responsive materials

**DOI:** 10.1107/S2052252524006055

**Published:** 2024-06-27

**Authors:** Gonzalo Campillo-Alvarado

**Affiliations:** a Department of Chemistry Reed College Portland OR 97202-8199 USA

**Keywords:** soft crystals, single-crystal-to-single-crystal transformations, dynamic crystals, chromism

## Abstract

The development of smart, stimuli-responsive materials has received increased attention in the past decade for their applications as sensing technologies. This commentary discusses a timely topical review by Kato [(2024). *IUCrJ*, **11**, 442–452] on the fabrication of multi-stimuli responsive crystals comprised of luminescent platinum(II) complexes, which exhibit intriguing chromic phenomena in response to stimuli.

Smart materials and modern sensing technologies demand molecular systems capable of responding to external stimuli without damaging the structural integrity. Ideally, materials should be robust yet flexible to accommodate molecular changes that result from external stimuli. In the current issue of **IUCrJ**, a topical review by Kato (Kato, 2024 ▸) discusses a remarkable class of luminescent platinum(II) complexes that assemble into soft crystals. Soft crystals (*i.e.* crystalline materials easily deformed near room temperature) respond to external stimuli such as light, heat, moisture, or chemical vapors with structural transformations and phase transitions. Combined with the luminescent properties of platinum(II) complexes and metallophilic interactions (*i.e.* Pt···Pt contacts), the soft crystals display intriguing chromic behavior in response to stimuli. These materials hold exciting promise as design elements for sensors and probes.

Recent discoveries in soft and dynamic crystals have challenged the enduring perception that crystals are predominantly static and used solely to determine molecular structures via X-ray diffraction (Naumov *et al.*, 2020 ▸; Kato & Ishii, 2023 ▸). Indeed, a plethora of recent reports of single-crystal-to-single-crystal (SCSC) transformations and other mechanical effects have demonstrated that single crystals and solids can be far from static. Mechanical effects have been broadly categorized into disintegrative and restorative (Awad *et al.*, 2023 ▸). Disintegrative effects cause crystals to break down after external stimuli in a one-time event, often leading to separation accompanied by a leaping or ‘salient’ effect. Conversely, cooperative and concerted molecular motions result in restorative effects, which enable crystals, including soft crystals, to retain macroscopic integrity, gain reversibility, and establish structure–property relationships by X-ray techniques (Posner *et al.*, 2015 ▸). Despite significant progress in the study of soft and dynamic crystals, establishing clear design elements and routes has long remained a major challenge in the field of crystal engineering, often relying on serendipity. The pertinent topical review by Kato (Kato, 2024 ▸) summarizes collective and systematic efforts on the development of Pt(II) complexes to generate resilient, adaptive and luminescent chromic crystals. These crystals respond to alterations in the molecular assembly with corresponding changes in color and luminescence, *i.e.* chromism (see Fig. 1 ▸). The review highlights chromism arising from alterations in the geometries of π···π stacking and Pt···Pt contacts caused by vapor exposure, mechanical stimuli, heating and counterion engineering.

A key element in the review by Kato (Kato, 2024 ▸) relies on discussing changes to the molecular structures in crystals arising from external stimuli by single-crystal X-ray diffraction (SCXRD). SCSC transformations and phase changes provide unique insights into chemical transformations in solids and help correlate with chromic luminescent effects (Posner *et al.*, 2015 ▸; Atwood *et al.*, 2002 ▸; Chaudhary *et al.*, 2017 ▸; Naumov & Bharadwaj, 2015 ▸). For instance, SCSC transformations have been used to understand molecular underpinnings of photochemical reactions and polymorphic transformations in pharmaceutics and organic semiconductors (Pettersen *et al.*, 2020 ▸; Chung & Diao, 2016 ▸; Sherman *et al.*, 2020 ▸). In the review, Kato discusses the relationships between Pt···Pt distances and emission color changes using variable-temperature SCXRD and emission spectra (Saito *et al.*, 2020 ▸). The results showed a high dependency of color on stacking patterns and Pt···Pt distances, which are further affected by the temperature changes and substituents in a series of Pt(II) complexes. The work is reminiscent of mechanical effects in crystals involving metallophilic interactions with gold (*i.e.* Au···Au contacts), which exhibit similar electronic and luminescent phenomena (Seki *et al.*, 2015 ▸).

The second part of Kato’s review discusses vapochromic transformations and triboluminescence in soft crystals of Pt(II) complexes, which are induced by vapor exposure and mechanical stimulus, respectively. Notably, single crystals exhibit numerous multi-stimuli transformations, allowing for cycling through vapor exposure, heating and grinding (Shigeta *et al.*, 2016 ▸) in a rare complex shape-memory behavior. Overall, Kato and collaborators have shown that SCSC transformations are an unparalleled analytic tool for understanding structure–property relationships of soft and dynamic crystals, as well as for developing advanced and reversible multi-functional materials.

Developing soft, dynamic crystals using Pt(II) complexes as building blocks, as discussed in the review by Kato (2024 ▸), represents a significant step forward for the rational and systematic design of stimuli-responsive chromic materials. Given the prominence of Pt(II) complexes in medicinal chemistry and catalysis, novel systems are expected to continue demonstrating unusual and attractive optical properties for materials design. Future development may include soft crystals using more than one metalophillic contact (*e.g.* Pt···Pt, Au···Au) or incorporating cooperative supramolecular interactions (*e.g.* chalcogen bonds, boron coordination). Advances could result in multi-responsive crystals capable of mechanical deformation and exhibiting reversible chromic phenomena in response to stimuli, potentially resulting in molecular-based sensors and devices that adjust to the curvature of surfaces such as human skin or plant leaves (Wang *et al.*, 2017 ▸; Wang *et al.*, 2024 ▸). The topical review by Kato (2024 ▸) is a clear and concise reference for crystal engineers and material scientists interested in developing a rational and systematic approach to designing multi-stimuli responsive soft crystals.

## Figures and Tables

**Figure 1:**
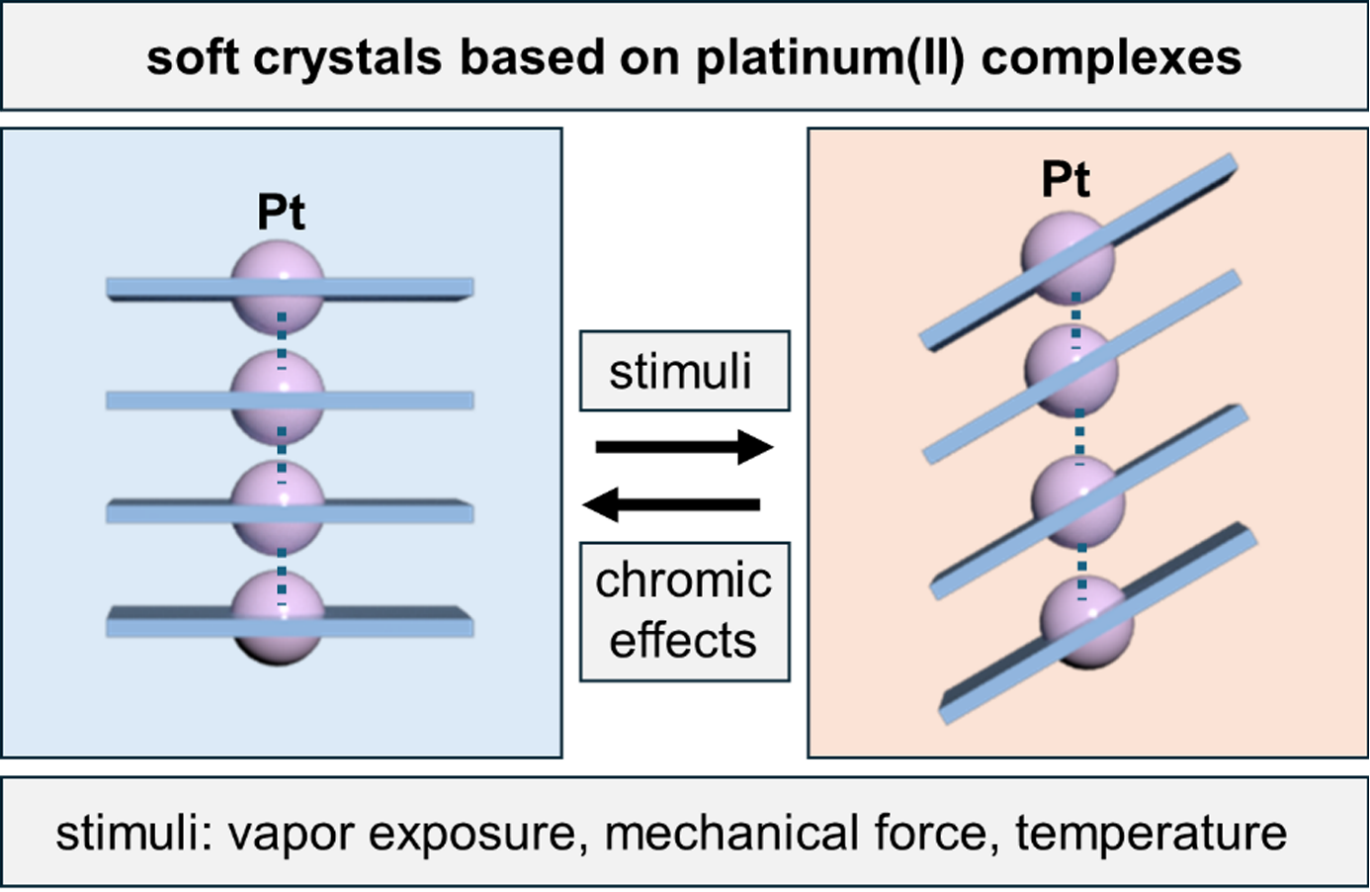
Reversible changes in color and luminescence in soft crystals of Pt(II) complexes triggered by gentle stimuli. Alterations in supramolecular architecture and interactions (π···π stacking and Pt···Pt contacts) generate changes in emission and optical phenomena.
